# Clinical Applications of Artificial Intelligence and Machine Learning in Children with Cleft Lip and Palate—A Systematic Review

**DOI:** 10.3390/ijerph191710860

**Published:** 2022-08-31

**Authors:** Mohamed Zahoor Ul Huqh, Johari Yap Abdullah, Ling Shing Wong, Nafij Bin Jamayet, Mohammad Khursheed Alam, Qazi Farah Rashid, Adam Husein, Wan Muhamad Amir W. Ahmad, Sumaiya Zabin Eusufzai, Somasundaram Prasadh, Vetriselvan Subramaniyan, Neeraj Kumar Fuloria, Shivkanya Fuloria, Mahendran Sekar, Siddharthan Selvaraj

**Affiliations:** 1 Orthodontic Unit, School of Dental Sciences, Health Campus, Universiti Sains Malaysia, Kubang Kerian, Kota Bharu 16150, Malaysia; 2 Craniofacial Imaging Lab, School of Dental Sciences, Health Campus, Universiti Sains Malaysia, Kubang Kerian, Kota Bharu 16150, Malaysia; 3 Faculty of Health and Life Sciences, INTI International University, Nilai 71800, Malaysia; 4 Division of Clinical Dentistry (Prosthodontics), School of Dentistry, International Medical University, Bukit Jalil, Kuala Lumpur 57000, Malaysia; 5 Orthodontic Division, Preventive Dentistry Department, College of Dentistry, Jouf University, Sakaka 72345, Saudi Arabia; 6 Prosthodontic Unit, School of Dental Sciences, Health Campus, Universiti Sains Malaysia, Kubang Kerian, Kota Bharu 16150, Malaysia; 7 Department of Biostatistics, School of Dental Sciences, Health Campus, Universiti Sains Malaysia, Kubang Kerian, Kota Bharu 16150, Malaysia; 8 National Dental Center Singapore, 5 Second Hospital Avenue, Singapore 168938, Singapore; 9 Faculty of Medicine, Bioscience and Nursing, MAHSA University, Kuala Lumpur 42610, Malaysia; 10 Faculty of Pharmacy, AIMST University, Bedong 08100, Malaysia; 11 Department of Pharmaceutical Chemistry, Faculty of Pharmacy and Health Sciences, Royal College of Medicine Perak, Universiti Kuala Lumpur, Ipoh 30450, Malaysia; 12 Faculty of Dentistry, AIMST University, Bedong 08100, Malaysia

**Keywords:** artificial intelligence, machine learning, diagnostic performance, treatment prediction, cleft lip and palate

## Abstract

Objective: The objective of this systematic review was (a) to explore the current clinical applications of AI/ML (Artificial intelligence and Machine learning) techniques in diagnosis and treatment prediction in children with CLP (Cleft lip and palate), (b) to create a qualitative summary of results of the studies retrieved. Materials and methods: An electronic search was carried out using databases such as PubMed, Scopus, and the Web of Science Core Collection. Two reviewers searched the databases separately and concurrently. The initial search was conducted on 6 July 2021. The publishing period was unrestricted; however, the search was limited to articles involving human participants and published in English. Combinations of Medical Subject Headings (MeSH) phrases and free text terms were used as search keywords in each database. The following data was taken from the methods and results sections of the selected papers: The amount of AI training datasets utilized to train the intelligent system, as well as their conditional properties; Unilateral CLP, Bilateral CLP, Unilateral Cleft lip and alveolus, Unilateral cleft lip, Hypernasality, Dental characteristics, and sagittal jaw relationship in children with CLP are among the problems studied. Results: Based on the predefined search strings with accompanying database keywords, a total of 44 articles were found in Scopus, PubMed, and Web of Science search results. After reading the full articles, 12 papers were included for systematic analysis. Conclusions: Artificial intelligence provides an advanced technology that can be employed in AI-enabled computerized programming software for accurate landmark detection, rapid digital cephalometric analysis, clinical decision-making, and treatment prediction. In children with corrected unilateral cleft lip and palate, ML can help detect cephalometric predictors of future need for orthognathic surgery.

## 1. Introduction

Cleft lip and palate (CLP) are one of the most common congenital deformities of craniofacial malformation leading to various dental anomalies in early childhood. Cleft lip and palate is the non-union of the upper lip and roof of the mouth; It may occur with a significant change in the shape and extent of congenital defects. The occurrence of CLP differs with various factors like race, ethnicity, geographical area, socioeconomic lifestyle, and type of cleft. The highest prevalence rate (1 in 500) has been reported in the Asian and American population [1]. The unilateral CLP is commonly found on the left side when compared to other side and is more common in males than females with a ratio of 2:1. Vander Woude syndrome, with an incidence of 1 in 70,000, is one of the most prevalent autosomal dominant diseases correlated to CLP or CP. This accounts up to 1 percent of all cases of syndromic CLP [2]. 

The etiology of non-syndromic CLP is still poorly understood. However, the origin of CLP is multifactorial; both environmental and genetic factors play a crucial role at certain points during the growth of the face [2,3,4,5]. Oral clefts are often associated with soft tissue, skeletal, and dental abnormalities. Discontinuity of the lip, alveolar process, missing or malformed teeth, and skeletal deformity in three planes (anteroposterior, vertical, and transverse) are examples of such defects [6]. Individuals with CLP may have congenitally or developmentally missing teeth [7]. The scar tissues in the palatal area of CLP patients not only affects oral hygiene but also alters the transverse and sagittal growth of the maxilla [8,9]. As a result, there is a subsequent decrease in the transverse dimension of the arch, especially in the anterior region [9]. The non-syndromic cleft lip and palate with or without palate (NSCL-P) is a major health concern that has an impact on affected individuals and their families’ quality of life, socioeconomic status, and psychological well-being. However, preventative measures have largely focused on raising awareness of potential environmental risk factors, such as drinking alcohol and smoking during pregnancy, and prescribing folic acid or multivitamin supplements, typically after conception. As no genetic counselling test has been established yet that can correctly predict the chance of couples having a child with NSCL-P, identifying predictive genetic risk factors for this condition is crucial [10]. 

The cleft disturbs the structural integrity of the palate, causing the minor portion of the maxilla to rotate medio-lingually and it is thought to be caused by the molding effect of the surrounding facial soft tissues which often results in a constricted palatal arch and severe anterior crossbite with or without posterior crossbite on the cleft side [11,12]. There are variety of methods for evaluating the craniofacial system, maxillary morphometry, dental relationship, and characteristics of CLP. Individual CLP measurements have already been shown by previous studies [13,14,15]. The findings of CLP’s craniofacial traits can be evaluated based on several parameters, for example, dental arch relationship [16], cephalogram [17,18,19], maxillary morphometry [20] and cone-beam computed tomography (CBCT) [21].

Artificial intelligence (AI) can extract information from a large amount of healthcare data using sophisticated algorithms, and then apply what it has learnt to improve clinical practices. Physicians may benefit from AI programs that supply up-to-date medical knowledge from journals, textbooks, and clinical procedures in order to assist them in providing appropriate patient care. Additionally, an AI device could help to reduce diagnostic and therapeutic errors that are unavoidable in human clinical settings. Furthermore, an AI device captures useful data from a broad patient population to aid in real-time inferences for health risk alarms and health outcome prediction [22,23]. 

Traditional aspects of dentistry are being modernized by AI. Convolutional neural networks (CNNs) and artificial neural networks (ANNs) are used in most published research (ANNs). In dentistry, AI has mostly been utilized to increase diagnostic accuracy and efficiency, which is crucial in achieving the greatest results from procedures while still offering exceptional care. When making a diagnosis and selecting the appropriate course of action, dentists must use all their knowledge in order to make accurate clinical decisions, they must also be able to predict the prognosis. Machine learning (ML) is a subset of AI that uses algorithms to forecast results based on a dataset. The main goal of ML is to enable machines to learn from data so they can solve problems without much human involvement [24]. 

### 1.1. Rationale and Objectives

Many studies have assessed the craniofacial dimension of CLP patients at the completion phase of the facial growth, whereas only a few studies have reported a deficiency in facial growth before the end of the growth stage [25,26,27]. The concave facial profile seen in cleft patients is caused by a sagittal defect of the midface, which is progressive and can be seen from childhood until adulthood [28]. However, careful evaluation from birth to adolescent age group is often required. As AI is frequently used in dentistry to construct automated software programs that simplify the diagnosis and data management [29], the AI models can be applied for precise diagnosis, clinical decision-making, and automatic cephalometric landmark detection [30].

Hence, the purpose of this systematic review was to (a) to explore the current clinical applications of AI/ML techniques in diagnosis and treatment prediction in children with CLP, and (b) to create a qualitative summary of results of the studies retrieved.

### 1.2. Research Questions

1. What are the current clinical applications of deep learning/artificial intelligence in patients with CLP?
2. What is the diagnostic performance of AI and ML models being utilized on CLP patients?

## 2. Materials and Methods

### 2.1. Research Design

The study followed the Preferred Reporting Items for Systematic review and Aeta-analysis (PRISMA) guidelines in the 2020 checklist and was successfully registered with the PROSPERO ID. CRD42021270601, but used a narrative-based research studies approach to summarize the literature. 

### 2.2. Eligibility Criteria

#### 2.2.1. Inclusion Criteria

− The articles that dealt with AI and its application in the context of CLP.
− The journal articles which present some predictability or observable outcomes using Machine learning techniques in children with CLP.
− Original articles, Case-control studies, longitudinal observational studies, and retrospective cross-sectional studies that involves artificial intelligent or machine learning neural network methods in children with CLP.

#### 2.2.2. Exclusion Criteria

− Unpublished articles that have been uploaded with only manuscripts.
− Articles that contain only abstracts without their full text.
− Journal articles which were published in languages other than English.
− Book chapters, magazine prints, blog posts, editorials, case reports and case series.

### 2.3. Information Sources

An electronic search was performed using the databases PubMed, Scopus, and Web of Science Core Collection. Two reviewers searched the databases separately and concurrently. The initial search was conducted on 6 July 2021. The publishing period was unrestricted; however, the search was limited to articles involving human participants and published in English. 

### 2.4. Search Strategy

The search keywords for each database were a combination of Medical Subject Heading (MeSH) phrases and free text terms. Across all databases, the vocabulary and syntax of terms were modified. Table 1 lists the search strings and keywords used with their respective databases.

### 2.5. Study Selection and Data Collection Process

The titles and/or abstracts of studies retrieved from the searches, as well as those retrieved from other sources (manual searching, reference/citation lists), were screened by two review authors to identify papers that may fulfil the inclusion criteria. The duplicates were removed using the Mendeley desktop (version 19.1.4) tool with check for duplicates option. One review author retrieved and read the full text of these potentially eligible papers, and any abstracts that were insufficiently detailed to allow decision-making. The decisions were double-checked by a second author. Any discrepancy between the two reviewers was discussed and resolved by the third author through mutual consent.

### 2.6. Data Extraction

The following information was extracted from the methodology and results parts of the papers that were chosen: the number and conditional characteristics of the AI training dataset that were used to train the intelligent system; the number and conditional characteristics of the machine learning classification models used to construct the intelligent system and the quantity of test data points utilized to evaluate the newly trained system against possible human comparisons and their learning outcomes.

### 2.7. Data Items

Data was sought based on the variables described as follows:

(a) Population—Children with Cleft lip and palate of either sex, and of any ethnicity.
(b) Intervention—The applications of AI/ML techniques in diagnosis and treatment prediction in children with CLP.
(c) Comparison—Human intelligence/other diagnostic methods which does not involve AI models.
(d) Outcomes—Diagnostic accuracy and prediction of treatment outcome in children with CLP.

### 2.8. Diagnostic Accuracy Measures

Accuracy (Ac) data was obtained as shown in the Table 2, while specificity (Sp) and sensitivity (Sn) were assessed. All results were standardized to a range of 0.00–1.00, and the normalized data was given a 1-point standard deviation [30]. The measurement is provided in the Supplementary File (S1).

### 2.9. Characteristics for Diagnostic Comparisons

The following criteria were used to further screen eligible publications and included research that made human versus machine diagnostic comparisons:

(i) Index test: the sensitivity and specificity of clinically trained AI/machine learning models are tested using an index test and evaluating parameters.
(ii) Reference standards: any other assessment techniques such as Mel frequency for hypernasality, lateral cephalometric radiographic evaluation by clinicians.
(iii) Target conditions: Unilateral CLP, Bilateral CLP, Unilateral Cleft lip and alveolus, Unilateral cleft lip, Hypernasality, Dental characteristics and sagittal relationship in children with CLP.

### 2.10. Risk of Bias Assessment

The Joanna Briggs Institute (JBI) Critical Appraisal Checklist for case-control studies was used to evaluate the possibility of bias among studies and potential discrepancies in the comparison [31].

### 2.11. Additional Synthesis

Due to the significant functional variations and clinical heterogeneity observed among the different disease classifications and machine learning models, a meta-analysis was deemed inappropriate. 

## 3. Results

### 3.1. Study Selection

**Figure 1:**
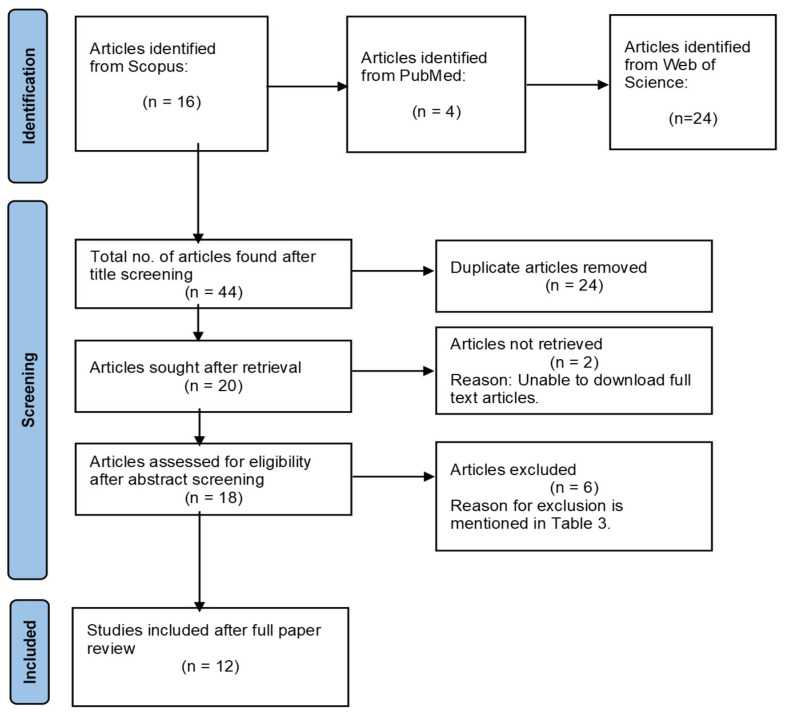
PRISMA flow diagram for studies searched.

A total of 44 articles was found in the Scopus, PubMed and Web of Science search based on the predefined search strings with accompanying database keywords. The PRISMA flowchart 2020 illustrates the initial search for articles screening and full paper reading with possible reasons for exclusion as shown in Figure 1. Following careful review of all articles, 24 duplicates were excluded, 8 articles were excluded, leaving 12 papers that were selected. Table 3 displays the reasons for exclusion.

### 3.2. Characteristics of the Included Studies

As per the inclusion criteria, we included 12 records [19,40,41,42,43,44,45,46,47,48,49,50] and their characteristics are listed in Table 4. These 12 studies discuss different clinical applications of AI/ML models in assessing their diagnostic performances in children with CLP.

### 3.3. Results of Risk of Bias Studies

Bias assessments were carried out independently by two reviewers. The likelihood of bias was rated as low when more than 70% of the responses were “yes,” moderate when 50% to 69% of the responses were “yes”, and high when up to 49% of the responses were “yes.” Studies with a moderate to high risk of bias were omitted from this review. Table 5 summarizes the results of Risk of bias assessment as per JBI critical appraisal checklist.

The risk of bias and its applicability concerns involving patient selection were rated as moderate to high due to inappropriate exclusion. The assessment of bias and applicability concerning the reference standard was rated as low because all studies used the clinician’s opinion as the reference standard. Regarding the bias risk and its applicability, referring to the index test was rated as low for all the studies included for assessment due to the consistent performance of deep learning methods [31].

### 3.4. Clinical Applications of AI

We divided the included studies into five categories: (i) Genetic risk assessment, (ii) Determining dental characteristics and sagittal jaw relationship, (iii) Detection of hypernasality, (iv) CLP surgery, and (v) Diagnosis and prediction of oral clefts. All the investigations were carried out in a single location, using data from the local population. Most of the research was done on speech evaluation in children with CLP. Most (4) studies were carried out on the Chinese population and few studies (2) were conducted on the Saudi Arabian population. The other studies being done on Brazilian, Iranian, Pakistani, and South American (3) children.

### 3.5. Genetic Risk Assessment

In individuals with non-syndromic cleft lip with or without palate (NSCL ± P), two studies looked at single nucleotide polymorphisms (SNPs) for diagnostic and predictive significance. The diagnostic ability of SNPs in Han and Uyghur populations has been validated by Zhang et al. [43]. Variations in two genes, methylenetetrahydrofolate reductase (MTHFR) and retinol-binding protein 4 (RBP4), were discovered to be crucial in the development of CLP. Machado et al. [42] investigated the Brazilian population for NSCL ± P and discovered interactions among the 13 SNPs involved, as well as a key function for genes involved in folate metabolism.

### 3.6. Dental Characteristics and Sagittal Jaw Relationship

One study evaluated the dental characteristics in children with different types of clefts in comparison with non-cleft individuals. Alam and Alfawzan [19] determined the dental characteristics between cleft and non-cleft subjects. Significant differences were observed among the various cleft groups when compared with the non-cleft group with highest accuracy rate of 94.5%. The sagittal jaw relationship between cleft and non-cleft individuals was evaluated. The AI driven lateral cephalometric analysis was performed using WebCeph software. When sagittal growth was compared between different types of clefts and NC individuals, the SNA, ANB angles, and Wits appraisal were all significantly reduced. The AI based lateral cephalometric assessment revealed 95.6% accuracy [44].

### 3.7. Hypernasality Detection 

Five research studies looked at how well the children with cleft lip and palate could recognize words. The most effective application of AI was in the assessment of hypernasality. Three studies attempted to detect it, that categorize hypernasality according to severity. The classifiers SVM, NN and DNN were used to extract the speech features as inputs. Detecting the presence of hypernasality was more accurate than determining the severity of hypernasality [40,45,46,47,48].

### 3.8. CLP Surgery

Deep learning technique was employed as a surgical assistance. Annotated frontal facial images were used to identify surgical markers in children with cleft lip and palate who were undergoing surgical repair of the cleft lip and palate. The aim was to limit the effect of the surgeon’s experience on the outcome [49].

### 3.9. Diagnosis and Prediction

A questionnaire was utilized in one study to collect information on 36 input characteristics from women, half of whom had babies with cleft lips and the other half were controls. Data was gathered and various forecasting models were used. The results obtained with each were assessed for accuracy. On test data, the MLP model with three hidden layers and 28 perceptron’s in each had the highest classification accuracy (92.6%) [50,51].

## 4. Discussion

In 1936, Alan Turing proposed the Turing machine, which can imitate the process of human calculation. The Turing machine concept, as well as the theory of computation provided a solid foundation for the Artificial intelligence research and development (AI). In 1956, after a period of twenty years, the phrase “artificial intelligence” was coined. The term AI was first defined in a Summer Research Project at Dartmouth as, “The study of any system that perceives its surroundings and takes steps to increase its chances of succeeding and attaining its objectives [52,53].

We conducted a systematic review to see how many AI applications are currently being used in the treatment of cleft lip and palate. Most (4) of the studies included in this systematic review were conducted in China, with at least one study from other nations such as Brazil, Saudi Arabia, Iran, Pakistan and three others in South America in each of the five categories. Although CLP is a common birth deformity, its prevalence differs among various ethnic origin. The greater prevalence of clefts among Asians compared to other ethnic groups suggests two probable reasons for the substantial research in China: the need to address a potential problem and the availability of data [54,55]. 

Single nucleotide polymorphisms (SNPs) were detected in studies that have investigated a genetic risk. These, too, were limited to a dataset gathered from specific groups, such as the Brazilian or Asian. In these studies, the application of machine learning was explored to predict the NSCL ± P genetic risk using a group of SNPs associated with NSCL ± P susceptibility which was previously identified in research with genome-wide association studies (GWAS) via linkage analysis that demonstrated strong evidence that a combination of SNPs are highly predictive in identifying the patients with NSCL ± P [42,43].

In studies to determine sagittal jaw relationship, authors have used the cephalometric radiographs for both cleft and NC individuals [56,57]. Although many researchers have recognized that atypical sagittal growth of the maxilla is a common manifestation in patients with UCLP, they have all concluded that the growth and direction of the jaw is completely influenced by the earlier treatment protocol, such as time and techniques of primary surgeries [58,59]. Hence, A.I.-driven automated lateral cephalometric analysis in such groups and populations has been utilized to avoid irrelevant estimating error and to make more precise, simple and quick radiographic interpretations for better treatment plans. These AI models have shown the greater accuracy rate of 94.5% [20,45].

The authors reported that restricted maxillary growth was often noted in children with surgically repaired UCLP [19,60]. In addition to postnatal treatment effects and congenital factors involved, altered craniofacial morphology was also seen in these children. As a result, the maximum alterations in different DC were found in patients with CLP in relation to NC individuals.

The hypernasality detection performance was found to be significantly higher in CLP group [46,47,48]. All ML techniques used in the included studies revealed the higher performance rate. These algorithms provide encouraging experimental results. Hypernasality identifying phonemes and misarticulations are used to define speech. The assessment of speech was based on extracted features and included the most studies possible. The input features are viewed as separate vectors among voice frames by the shallow and direct neural network (DNN) classifiers. The studies involved in this review presented a hypernasal speech detection system based on the CNN and long short-term memory—direct recurrent neural network (LSTM-DRNN), which not only mines deep feature information through a multi-layer vertical connection, but also collects short-time dependencies between speech frames through horizontal parameter sharing. Another study utilized the NLD and entropy measures taken from the reconstructed attractor. Following this, the most important features were selected based on the principal component analysis (PCA), and the decision on whether or not to use a voice record that is hypernasal or healthy was taken with an SVM. The suggested hypernasality detection method based on LSTM-DRNN achieved the highest accuracy of 93.35%.

More information, both in terms of quantity and variance, will help a model make more accurate predictions. These predictive algorithms, such as one that may determine pathway of cleft formation via exposure to toxic, can be implemented as public health tools to prevent cleft development or raise awareness among the local community when prevention is not possible [61].

The limitation of this systematic review, though we tried to be as thorough as possible by including artificial intelligence and machine learning as a MeSH term, it is possible that more specific search terms like neural networks, support vector machines, and supervised or unsupervised learning, could have provided additional results.

## 5. Conclusions

Artificial intelligence is an advanced technique that can be used for precise landmark identification, rapid digital cephalometric analysis, clinical decision-making, and treatment prediction using AI-enabled programming software. The AI method has also been applied in pre-surgical orthopedics, speech pathology detection, and need for prediction of CLP surgery. In controlled circumstances, the models produced so far have shown great potential with higher accuracy rates from 85 to 95.6%. and found to be good performance of these ML models. However, their applicability cannot be generalized because they have not been prospectively tested in different clinical settings. Hence, longitudinal studies with multi-center trials are required to validate these AI models in future. In children with corrected unilateral cleft lip and palate, ML can help detect cephalometric predictors of future need for orthognathic surgery. Indeed, it was believed that, despite future advances in AI, it is not possible to replace human logic, rather than using it to support the decision of human clinicians.

## Figures and Tables

**Table 1:** Search strategy and keywords strings.

Nos	Keyword Strings	Results Obtained in Scopus (S)	Results Obtained in PubMed (P)	Results Obtained in Web of Science (W)	Articles Screened from Results According to Title (S + P + W)
1	Craniofacial anomaly + Oral clefts * + Artificial intelligence *	0	2	0	02
2	Artificial intelligence * + Cleft lip and palate * + automated landmarks	01	0	01	02
3	Oral cleft * + Machine learning * + prediction	02	0	03	05
4	Neural network * + Deep learning * + Cleft lip and palate *	03	0	06	09
5	Machine learning * + clefts * + sagittal relationship	01	0	0	01
6	Machine learning * + Genetic risk + Oral clefts *	01	01	03	05
7	Artificial intelligence * + anatomical variations + Cleft lip and palate *	0	0	0	0
8	Automatic detection + hypernasal speech + Cleft lip and palate *	05	0	06	11
9	Cleft Lip and Palate * + Surgery + Deep learning *	02	01	02	05
10	Facial morphology + oral clefts * + Machine learning *	0	0	0	0
11	Maxillofacial defect + Machine learning * + orofacial clefts *	0	0	0	0
12	Speech recognition + Artificial intelligence * + Oral clefts *	0	0	0	0
13	Artificial intelligence * + Orthognathic surgery + Prognostics factors	01	0	01	02
14	Artificial intelligence * + Dental characteristics + clefts *	0	0	02	02
	Total	16	04	24	44

String asterisk (*) was used to search all the possible words along with them, S = Articles from Scopus database for each string, P = Articles from PubMed database, W = Articles from Web of Science database for each string.

**Table 2:** Sensitivity and Specificity assessment for diagnostic accuracy.

Test outcome (index test)		Disease status (reference standard result)
True positives (a)	False positives (b)	Test positives (a + b)
False negatives (c)	True negatives (d)	Test negatives (c + d)
Index test positive (T+)		Index test negative (T−)

**Table 3:** Articles excluded and reason for exclusion after reading the full paper.

Author Name with Year of Publication	Title of the Article	Reason for Exclusion
Orozco-Arroyave et al. [32]	Characterization methods for the detection of multiple voice disorders: Neurological, functional, and laryngeal diseases	The authors did not use any of the AI or machine learning techniques in this study.
Dubey et al. [33]	Detection and assessment of hypernasality in repaired cleft palate speech using vocal tract and residual features	The authors used different methods for detection and assessment of hypernasality in children with CLP but no AI or machine learning methods involved in the study.
Phan et al. [34]	Tooth agenesis and orofacial clefting: genetic brothers in arms?	This is a review paper on tooth agenesis and orofacial clefting based on genetic loci but did not mention about any AI models.
Mathiyalagan et al. [35]	Meta-Analysis of Grainyhead-Like Dependent Transcriptional Networks: A Roadmap for Identifying Novel Conserved Genetic Pathways	The meta-analysis was done to identify the genes causing oral clefting but no AI or Machine learning techniques used in this study
Lim et al. [36]	Determination of prognostic factors for orthognathic surgery in children with cleft lip and/or palate	Unable to download the full content of this study.
Carvajal-Castaño and Orozco-Arroyave, [37]	Articulation Analysis in the Speech of Children with Cleft Lip and Palate	This article is a chapter from the book “Progress in Pattern Recognition Image Analysis, Computer Vision and Applications”.
Zhang et al. [38]	Cleft Volume Estimation and Maxilla Completion Using Cascaded Deep Neural Networks	This paper is a chapter from the book “Machine Learning in Medical Imaging”.
Tanikawa et al. [39]	Clinical applicability of automated cephalometric landmark identification: Part I—Patient-related identification errors	Unable to download the full text article.

**Table 4:** Characteristics and Methodology of the included studies.

Author	Target Condition	Sample Size	AI Technique and Method Employed	Findings
Machado et al. [42]	Genetic risk assessment in non-syndromic CLP	722 Brazilian subjects with NSCL ± P and 866 without NSCL ± P	RF and multi-layer NN. The genetic risk of NSCL ± P in the Brazilian population was developed by putting 72 known SNPs to RF, which was then used to identify important SNPs. Multiple regression was used to assess the interactions between the SNPs.	13 SNPs were found to be highly predictive to detect NSCL ± P. The combination of these SNPs was able to split the controls from NSCL ± P with highest accuracy rate of 94.5%.
Zhang et al. [43]		504 East asians,103 Han Chinese and 279 Uyghur Chinese with CLP	SVM, LR, NB, DT, RF, k-NN, and ANN. Machine learning techniques were used to validate the diagnostic ability of 43 SNP candidates in assessing genetic risk in Chinese populations. After manual selection, a panel of 24 SNPs was assessed for risk assessment efficiency. Each time the LR-based model was trained, an SNP was removed or added in a sequential manner.	In the Han population, the LR model produced the greatest results for genetic risk assessment, whereas the SVM produced better results in the Uyghur group. The relative risk score methodology produced the greatest results in the Uyghur population. SNPs in three genes involved in folic acid and vitamin A production were found to play a critical role in the occurrence of NSCL ± P.
Alam et al. [44]	Sagittal jaw relationship in cleft and non-cleft individuals	123 Saudi Arabian patients 21 BCLP, 41 UCLP, 13 UCL, 9 UCLA and 31 NC individuals	AI driven WebCeph software. The LCRs of patients were used to measure 4 different parameters such as SNA, SNB, ANB and Wits appraisal.	The comparison of sagittal development among different types of clefts with NC subjects revealed significant smaller SNA, ANB angles and Wits appraisal. However, there was no significant variation observed in SNB angle between cleft and non-cleft subjects. Also, there was no significant difference found in terms of gender and types of clefts.
Alam and Alfawzan [19]	Dental characteristics in cleft and non- cleft individuals	123 Saudi Arabian subjects 92 cleft and 31 non-cleft individuals	AI driven lateral cephalometric analysis was done using WebCeph software. 14 different dental characteristics such as OJ, OB U1 to FH, U1 to SN U1 to UOP, IMPA L1 to LOP, IIA, COP U1 to NA (mm), U1 to NA (degree), L1 to NB (mm), L1 to NB (degree), UID were evaluated.	Significant disparities among cleft and NC subjects were found in relation to Overjet, U1 to FH, U1 to SN, U1 to IMPA, IIA, U1 to NA (degree) and L1 to NB (degree). However, no significant differences were observed between cleft and NC in relation to OB, U1 to UOP, L1 to LOP, COP, U1 to NA (mm), L1 to NB (mm) and UID. AI based cephalometric assessment showed 95.6% accuracy.
Wang et al. [45]	Detection of Hypernasality in cleft palate patients	144 Chinese patients (72 with hypernasality and 72 controls)	LSTM-DRNN method which is used for automatic detection of hypernasal speech, vocal cords related feature mining, classification ability and analysis of hypernasality- sensitive vowels.	LSTM-DRNN achieved highest 91.10% accuracy in automatic hypernasal speech detection compared with shallow classifiers. The GD spectrum and PSD have shown 93.35% and 90.26% accuracy, respectively.
Golabbakhsh et al. [46]		15 CLP patients and 15 controls (Iranian population)	SVM. Automatic detection of hypernasality with acoustic analysis of Speech. Mel frequency, bionet wavelet transform entropy.	When combined with SVM, Mel frequency and bionet wavelet transform energy 85% of the accuracy have been achieved in identifying hypernasality.
Wang et al. [47]		62 Children and 48 adults (Chinese patients)	CNN. Hypernasality detection.	A hypernasality detection accuracy of 93.34% was achieved with CNN compared with state-of-the-art literature.
Orozco-Arroyave et al. [48]		South American children with CLP	SVM. Automatic identification of hypernasal speech of Spanish vowels using classical and non-linear analysis	The NLD analysis provide relevant information and can be used as an alternative classical Mel frequency in automatic detection of hypernasality in Spanish vowels. The greater accuracy of 95.4% was achieved with only NLD features.
Orozco-Arroyave et al. [40]		Spanish subjects Cases 130 Controls 108 German subjects Cases 429 Controls 39	A SVM was used to determine whether a voice recording is hypernasal or healthy.	It was found that the combination of NLD features and entropy measurements yield best results. The addition of information provided by the five vowels in the discriminating process results in an improvement in system performance for each vowel.
Mathad et al. [41]		75 cases 251 controls (American population)	A DNN classifier was created to distinguish between nasal and non-nasal speech sounds using a healthy voice corpus.	The proposed DNN method employs forced-alignment, which could lead to incorrect segmentation and impact the hypernasality estimator’s effectiveness.
Li et al. [49]	Cleft lip and palate surgery	2568 CLP cases (Chinese population)	Deep learning technique for CLP surgery. Train the model to locate surgical incisions and markers. State-of-the-art Hour glass architecture and residual learning models were used to create strong baseline dataset.	CLPNet-Light and VGG are significantly better than two CSR-based techniques. The CLPNet-Light is 2.5 times higher than CLPNet which has strong robustness and can be used to train the model to aid in surgical marker localization.
Shafi et al. [50]	Prediction of oral cleft	1000 Pakistani subjects (500 cases and 500 controls)	DNN. A questionnaire was designed to collect information on 36 input characteristics from mothers, half of whom had cleft babies and the other half were controls. Data was gathered and various prediction models were used. The precision of the results obtained with each were assessed.	On test data, the MLP model with three hidden layers and 28 perceptrons in each provided the highest classification accuracy rate of 92.6%.

**Table 5:** The summary results of Risk of bias assessment as per JBI critical appraisal checklist.

No	Authors	Country	Study Design	Sample Size (*n*)	Quality Assessment (%)	Risk of Bias Rating
1	Machado et al. [42]	Brazil	Retrospective Case control	1588	90.0	LOW
2	Zhang et al. [43]	China	Retrospective Case control	171	90.0	LOW
3	Alam et al. [44]	Saudi Arabia	Retrospective Case control	123	80.0	LOW
4	Alam and Alfawzan [19]	Saudi Arabia	Retrospective Case control	123	80.0	LOW
5	Wang et al. [45]	China	Retrospective Case control	144	60.0	MODERATE
6	Golabbakhsh et al. [46]	Iran	Retrospective Case-control	30	80.0	LOW
7	Wang et al. [47]	China	Retrospective Case control	110	80.0	LOW
8	Orozco-Arroyave et al. [48]	South America	Retrospective Case control	238	80.0	LOW
9	Orozco-Arroyave et al. [40]	South America	Retrospective Case control	202	90.0	LOW
10	Mathad et al. [41]	South America	Retrospective Case control	326	50.0	HIGH
11	Li et al. [49]	China	Retrospective	2568	50.0	HIGH
12	Shafi et al. [50]	Pakistan	Prospective	1000	70.0	LOW

## Data Availability

Not applicable.

## Supplementary Materials

The following supporting information can be downloaded at: https://www.mdpi.com/article/10.3390/ijerph191710860/s1, Table S1: Sensitivity and Specificity assessment for diagnostic accuracy.

## Abbreviations

ANB	A-point, nasion, B-point
ANN	Artificial neural network
BCLP	Bilateral CLP
CLP	cleft lip and palate
CNN	Convolutional neural network
COP	Cant of occlusal plane
CSR	Cascaded shaped regression
DNN	Deep neural network
DRNN	Deep recurrent neural network
DT	Decision tree
FH	Frankfort horizontal
GD	Group display
IIA	Inter-incisal angle
IMPA	Incisor mandibular plane angle
k-NN	k-nearest neighbor
L1	Lower central incisor
LOP	Lower occlusal plane
LR	Logistic regression
LSTM	Long short-term memory
MLP	Multi-layer perceptron
NA	Nasion to point-A
NB	Naive Bayesian
NB	Nasion to point-B
NC	Non-cleft
NLD	Non-linear dynamics
NSCLP ± P	Non-syndromic cleft lip and palate with or without palate
OB	Overbite
OJ	Overjet
PSD	Power spectrum density
RF	Random forest
SN	Sella nasion
SNA	Sella, nasion, A-point
SNB	Sella, nasion, B-point
SNPs	Single nucleotide polymorphism
SVM	Support vector machine
U1	Upper central Incisor
UCL	Unilateral cleft lip
UCLA	Unilateral cleft lip and alveolus
UCLP	Unilateral CLP
UID	Upper incisor display
UOP	Upper occlusal plane,
VGG	Visual Geometry Group
