# Supplementary material for: Clinical Applications of Artificial Intelligence and Machine Learning in Children with Cleft Lip and Palate—A Systematic Review
Source: Int J Environ Res Public Health. 2022 Aug 31;19(17):10860. doi: 10.3390/ijerph191710860 (PMC9518587; doi:10.3390/ijerph191710860)
Supplement: Supplementary file 1 [file ijerph-19-10860-s001.zip › ijerph-1867032-supplementary/ijerph-1867032-Table S1. Sensitivity and Specificity assessment for diagnostic accuracy.pdf]

Disease status (reference standard result)

Test outcome (index test)	Diseased (D+)	Non-diseased (D-)	Total
Index test positive (T+)	True positives (a)	False positives (b)	Test positives (a+b)
Index test negative (T-)	False negatives (c)	True negatives (d)	Test negatives (c+d)
Total	Disease positives (a+c)	Disease negatives (b+d)	N (a+b+c+d)

Author	Target condition	Testing sample size (N)	Reference standard TP	FP	FN	TN	Accuracy (%)
Zhang et al., 2018	Genetic risk	1587	504	205	382	496	92.6
Machado et al., 2021		2266	722	207	515	822	94.5
Alam et al., 2021	Sagittal relationship	154	92	31	0	31	94.5
Alam and Alfawzan	Dental characteristics	154	92	31	0	31	95.6
Golabbakhsh et al. 2017	Hypernasality	45	15	15	0	15	85
Orozco Arroyave et al., 2012		706	130	429	39	108	German 88 Spanish 95
Wang et al., 2019		223	62	48	48	65	93.34
